# Critical resistance to carbapenem and aminoglycosides in *Pseudomonas aeruginosa*: spread of *bla*_NDM_/16S methylase *armA* harboring isolates with intrinsic resistance mechanisms in Kerman, Iran

**DOI:** 10.1186/s12879-024-10085-w

**Published:** 2024-10-21

**Authors:** Behnaz Soltani, Roya Ahmadrajabi, Davood Kalantar-Neyestanaki

**Affiliations:** 1 Medical Mycology and Bacteriology Research Center, Kerman University of Medical Sciences, Kerman, Iran; 2 Department of Medical Microbiology (Bacteriology and Virology), Afzalipour Faculty of Medicine, Kerman University of Medical Sciences, Kerman, Iran

**Keywords:** *Pseudomonas aeruginosa*, Biofilm, Carbapenemase, AmpC β-lactamase, Efflux pump, *oprD*

## Abstract

**Background:**

Carbapenem-resistant *Pseudomonas aeruginosa* (CRPA) is one of the main Gram-negative bacterium causes of infections in hospital settings, and the spread of them is a significant challenge to public health.

**Methods:**

A total of 30 non-duplicate isolates of CRPA were collected. Antibacterial susceptibility of isolates to antibiotic agents, AmpC β-lactamase production, and biofilm formation were determined. Minimum biofilm inhibitory concentrations (MBIC) of isolates to cefepime (FEP), imipenem (IPM), ceftazidime (CAZ), and meropenem (MEM) were evaluated with/without cloxacillin (CLX). The carbapenemase and 16 S rRNA methylase genes were identified by PCR, and the transcription levels of *oprD*, *ampC*, and *mexA* genes were determined by quantitative real-time PCR (qPCR). ERIC-PCR was used to detect genetic relationships among isolates.

**Results:**

All isolates were multidrug resistant (MDR) and strong biofilm producers. The resistance genes including *bla*_NDM_, *bl*a_IMP_, *bla*_VIM_, *bla*_SIM_, *bla*_GES,_ and *armA* were detected in 21 (70%), 6 (20%), 3 (10%), 2 (6.6%), 1 (3.3%), and 17 (56.6%) of the isolates, respectively. CLX at 500 and 1000 µg/mL significantly reduced the level of MIC to MEM, IPM, CAZ, and FEP, also at 2000 µg/mL significantly reduced the level of MBIC to MEM, IPM, CAZ, and FEP. In all isolates, the transcription levels of *oprD* were significantly downregulated as well as significantly increased for *ampC* and *mexA*. ERIC-PCR typing results divided 30 isolates into four clusters A to D.

**Conclusion:**

In this study, we reported the spread of different clones of CRPA harboring co-existence of various carbapenemase genes with *armA* 16 S rRNA methylase for the first time in Kerman, Iran. Also, our isolates had several mechanisms of resistance to carbapenems as well as ability biofilm formation along with resistance to aminoglycosides, the further spread of which could cause serious challenges in our hospital settings. Therefore, serious monitoring is necessary to reduce their prevalence.

## Background

The effective management of severe hospital infections caused by Gram-negative bacterial pathogens is becoming progressively challenging due to the ongoing depletion of available treatment options [1]. *Pseudomonas aeruginosa* (*P. aeruginosa*) is one of the main opportunistic Gram-negative bacilli with extensive intrinsic resistance to multiple antibiotics; infections by this bacterium are a major challenge in hospital settings, especially in patients with compromised immune systems [2, 3]. One of the most notable characteristics exhibited by this pathogen is its capacity to develop resistance to antibiotic agents through chromosomal mutations, acquire new resistance genes as well as biofilm forming [4]. Carbapenems, along with other antibiotics such as aminoglycosides, are one of the most important therapeutic options for infections caused by MDR *P. aeruginosa* [5]. However, with widespread resistance to these antimicrobial agents in *P. aeruginosa*, we have limitations in use of them for infection treatment by this bacterium [6]. Resistance to carbapenems by *P. aeruginosa* may occur through loss or mutation of the outer membrane porin such as OprD, overexpression of the efflux pumps, including MexAB-OprM, overproduction of AmpC β-lactamases, and biofilm formation as intrinsic resistance mechanisms [5]. In addition, *P. aeruginosa* has the ability to develop resistance to carbapenems by acquiring metallo-β-lactamases (MBLs) or carbapenemases through horizontal gene transfer (HGT) [7]. Carbapenemases are able to hydrolyze broad-spectrum β-lactam antibiotics, including the third and fourth generations of cephalosporins and carbapenems [8]. Recently, various carbapenemase genes such as *bla*_VIM_, *bla*_IMP_, *bla*_SPM_, *bla*_GIM_, *bla*_SIM_, *bla*_KPC_, *bla*_OXA−48_, and *bla*_NDM_ have been reported around the world in carbapenem-resistant clinical isolates of *P. aeruginosa* [7, 9].

Aminoglycosides are one of the antibiotic agents that may be used in combination with carbapenems in the treatment of severe infections by *P. aeruginosa* [10]. Similar to carbapenems, resistance to these antibiotics is increasing, and current studies have reported a novel plasmid-mediated aminoglycoside resistance mechanism caused by 16S rRNA methylase in clinical *P. aeruginosa* isolates [11]. This mechanism confers high levels of resistance to aminoglycosides and is usually coupled with carbapenemase genes [10]. As yet more than ten types of 16S rRNA methyltransferases, including *armA*, *rmtA* to *rmtH*, and *npmA*, have been reported among nosocomial pathogens [11, 12]. Biofilm formation during the infection plays an important role in the recurrence of infection, resistance to antibiotics, and immune system [13]. Eliminating biofilms often requires high and continued dosages of antibiotics, which is usually not successful [14]. In many cases, eradicating biofilm during infection requires a combination of antimicrobial therapies. Combining of antimicrobial agents is considered one potential strategy for eradication biofilm forming during infection treatment [13]. Molecular typing methods are important tools for detecting the source of nosocomial outbreaks and to implement effective control methods for the prevention the spread of resistant pathogens in hospital settings. Different molecular typing techniques, such as enterobacterial repetitive intergenic consensus-polymerase chain reaction (ERIC-PCR) and multilocus sequence typing (MLST), have been developed and used for epidemiological investigations of *P. aeruginosa*. Among the molecular typing techniques, ERIC-PCR generally used for short-term and local outbreaks [15].

MDR *P. aeruginosa* isolates can lead to serious infections, prolonged hospital stays, and poor patient outcomes due to treatment failure, so monitoring antibiotic resistance patterns, investigating the presence of resistance mechanisms, and the genetic relationship of hospital isolates is very necessary to use the appropriate treatment strategies and infection control. Therefore, the aim of this study was to determine the mechanisms of resistance to carbapenems and aminoglycosides, including expression levels of the *mexA*, *ampC*, and *oprD*, frequency of carbapenemase and 16S rRNA methylation genes, biofilm formation, as well as molecular typing of carbapenem-resistant *P. aeruginosa* isolates in Kerman, Iran. Also, we investigated the effect combination of imipenem, meropenem, ceftazidime, and cefepime with cloxacillin on inhibition of biofilm formation.

## Materials and methods

### Bacterial isolates

In the present study, during February to May 2022, 30 non-duplicated clinical isolates of carbapenem-resistant *P. aeruginosa* were collected from bronchoalveolar lavage (BAL) of hospitalized patients at Afzalipour hospital in the internal ward, Kerman, Iran. The isolates were identified as *P. aeruginosa* using biochemical tests including Gram staining, oxidase test, non-carbohydrate fermentation on MacConkey and triple sugar iron agar (TSI), pigman production, oxidative-fermentative (O/F) test, growth on cetrimide agar, and at 42 °C. After identification, the isolates were stored in tryptic soy broth (TSB) with 20% glycerol at -80 °C for more investigation [16]. All media in this study were provided from CONDA (Condalab Co., Spain) or HiMedia (Himedia Laboratories Pvt. Ltd., India).

### Antibacterial susceptibility tests

Antibacterial susceptibility of isolates was determined according to the Clinical & Laboratory Standards Institute (CLSI, M100; 2022) recommendations [17]. The disc diffusion method was used for assessing the antibacterial susceptibility of isolates to several classes of antibiotics (PADTAN TEB Co., Iran), including meropenem (MEM, 10 µg), imipenem (IPM, 10 µg), cefepime (FEP, 30 µg), ceftazidime (CAZ, 30 µg), gentamicin (GEN, 10 µg), tobramycin (TOB, 10 µg), and ciprofloxacin (CIP, 5 µg). Minimum inhibitory concentrations (MICs) of the isolates to IPM, MEM, CAZ, and FEP (Jaber Ebne Hayyan Pharmaceutical Co., Tehran, Iran) with/without 125, 250, 500 µg/mL of cloxacillin (CLX; Farabi Pharmaceutical Co., Isfahan, Iran) were used for detection of AmpC overproducer isolates. The isolates were considered an AmpC overproducer when there was at least a 2-fold dilution reduction between the MIC of CAZ, or one of IPM, MEM, and FEP plus CLX [16, 18]. *P. aeruginosa* ATCC 27853 and *E. coli* ATCC 25922 were used as standard strains in the antibacterial susceptibility tests.

### Biofilm production assay

We used the microtiter technique previously described by Stepanovic et al., using safranin staining in 96-well polystyrene microtiter sterile plates for the biofilm-forming assay [19]. *Pseudomonas aeruginosa* PAO1 was used as positive controls in this experiment, and clinical isolates were categorized into four levels of biofilm formation, including strong biofilm formation was characterized as OD > 4 × ODc; moderate biofilm formation was characterized as 2 × ODc < OD ≤ 4 × ODc; weak biofilm formation was characterized as ODc < OD ≤ 2 × ODc; and non-biofilm formation was defined as OD ≤ ODc.

### Determination MBICs of isolates to IPM, MEM, FEP, and CAZ with/without CLX

The minimum biofilm inhibitory concentrations (MBIC) of isolates to IPM, MEM, CAZ, and FEP with/without 250, 500, 1000, and 2000 µg/mL of CLX in isolates were determined using the broth microdilution method on 96-well polystyrene plates with a flat-bottom microplate shape. In a nutshell, a suspension of a bacterial isolate with an inoculum of 1 × 10^6^ CFU/mL was diluted in tryptic soy broth plus 1% glucose with a series of dilutions of CAZ, FEP, IPM, and MEM, with/without a constant concentration of one of the sub-MICs of CLX (250, 500, 1000, and 2000 µg/mL) and then incubated at 37 °C. After incubation for 24 h, the MBIC level was determined using safranin staining in the lowest concentration of antibiotic with/without CLX that resulted in OD_490nm_ difference at or below 10% of the mean of three positive growth-control well readings [20].

### Detection of carbapenemase and 16S rRNA methylase genes by PCR

The boiling method was used for genomic DNA extraction [21]. PCR was carried out in a 20 µL reaction containing 10 µL of 2X Taq DNA Polymerase Master Mix RED (AMPLIQON, Co., Denmark), 0.25 µL of each primer (Forward and Revers primer), 1 µL of DNA templates, and 8.5 µL of DNase and RNase-free sterile water (Tehran Cavosh Clon, Co., Iran) in a thermal cycler (Biometra, Co., Germany) for detection of carbapenemase genes including *bla*_IMP_, *bla*_VIM_, *bla*_SIM_, *bla*_SPM_, *bla*_GIM_, *bla*_KPC_, *bla*_GES_, *bla*_NDM_, and 16S rRNA methylase; *rmtA*, *rmtB*, *rmtC*, and *armA*. The primer sequences, annealing temperature, and PCR condition are presented in Table 1 [12, 15]. The PCR products were electrophoresed on a 1% agarose gel containing 4 µL safe DNA staining dye (Pars tous Biotechnology, Co., Iran) in 0.25× Tris-EDTA-boric acid buffer (TBE, Tehran Cavosh Clon, Co., Iran).

**Table 1:** The sequence of primers used in PCR and qPCR

Target genes	Sequence of Primer (5´-3´)	Product Size (bp)	Annealing (° C)	PCR and qPCR Conditions	Use	Reference
*bla*_*VIM*_	F-ATGTTAAAAGTTATTAGTAGT R-CTACTCGGCGACTGAGCGAT	391	56	5 min at 95 °C (1 cycle), 1 min at 95 °C, 1 min for annealing, and 1 min at 72 °C (30 cycles) and finally 10 min at 72 °C.	In PCR	[15]
*bla*_*IMP*_	F-GGAATAGAGTGGCTTAAYTCTC R-GGTTTAAYAAAACAACCACC	233	58			
*bla*_*NDM*_	F-GGTTTGGCGATCTGGTTTTC R-CGGAATGGCTCATCACGATC	621	59			
*bla*_*GIM*_	F-TACAAGGGATTCGGCATCG R-TAATGGCCTGTTCCCATGTG	477	59			
*bla*_*SPM*_	F-AAAATCTGGGTACGCAAACG R-ACATTATCCGCTGGAACAGG	271	59			
*bla*_*SIM*_	F-TACAAGGGATTCGGCATCG R-TAATGGCCTGTTCCCATGTG	571	58			
*bla*_KPC_	F-TCTGGACCGCTGGGAGCTGG R-TCGCCGTTGACGCCCAATCC	798	60			
*bla*_GES_	F-ATGCGCTTCATTCACGCAC R-CTATTTGTCCGTGCTCAGG	864	59			
*armA*	F-AAAGTACAATCAGGGGCAGTT R-TCGTCGTCTTTAACTTCCCAA	269	60			[12]
*rmtA*	F-CTAGCGTCCATCCTTTCCTC R-TTGCTTCCATGCCCTTGCC	634	59			
*rmtB*	F-GCTTTCTGCGGGCGATGTAA R-ATGCAATGCCGCGCTCGTAT	173	60			
*rmtC*	F-CGAAGAAGTAACAGCCAAAG R-ATCCCAACATCTCTCCCACT	711	56			
*ampC*	F-CGCCGTACAACCGGTGAT R-GAAGTAATGCGGTTCTCCTTTCA	81	60	15 min at 95 °C (1 cycle), 30 s at 95 °C, 30 s for annealing, and 30 s at 72 °C (40 cycles) and then performed melting curve analysis ranging temperature 95 to 60 °C	In qPCR	[22]
*rpoD*	F-TTGATCCCCATGTCGTTGATC R-ACCACCTGCCGGAGGATATTTCCGAT	148				
*mexA*	F-CAAGCAGAAGGCCATCCTC R-CGGTAATGATCTTGTCGCCG	182				[15]
*oprD*	F-TCCGCAGGTAGCACTCAGTTC R-AAGCCGGATTCATAGGTGGTG	191				[7]

### Gene expression experiment by qPCR

The transcriptional level of the *mexA*, *oprD*, and *ampC* genes of the isolates was determined by the standard curve method using relative qPCR. The *rpoD* was considered as reference gene for normalizing the transcriptional levels of *mexA*, *oprD*, and *ampC* as target genes using the Pfaffl equation. The genes were down- or up-regulated in clinical isolates when the level of RNA was at least 2-fold lower or higher than those in the PAO1 as a calibrator (reference strain). Briefly, clinical isolates were grown separately in 10 mL TSB medium (Condalab Co., Spain) in a shaker incubator at 37 °C and 180 rpm to the log phase (optical density at 600 nm [OD 600] = 0.8–1), and then bacterial cells were collected by centrifugation at 12,000 rpm for 5 min. Total RNA of the isolates was extracted using TRIzol (ASAGENE, Co., Iran). Residual and DNA contamination was removed by treatment with the RNase-Free DNase I (SinaClon, Co., Iran), and cDNA was synthesized using a Transcriptor First Strand cDNA Synthesis Kit (AddBio, Co., South Korea) according to the manufacturer’s recommendations. The qPCR experiment was carried out in RealQ Plus 2x Master Mix Green (AMPLIQON, Co., Denmark) using StepOnePlus Real-Time PCR Systems (Applied Biosystems, Co., USA) in triplicate with both negative and positive controls in an amount of 20 µL containing 10 µL RealQ Plus 2x Master Mix Green, 0.5 µL cDNA, 0.5 µL of each primer, and 8.5 µL DNase and RNase-free sterile water (Tehran Cavosh Clon, Co., Iran). The primer sequences, annealing temperature, and qPCR condition in this study are presented in Table 1 [7, 15, 22].

### Molecular typing by ERIC-PCR

The ERIC-PCR was performed using ERIC1 (5´-ATGTAAGCTCCTGGGGATTCAC-3´) and ERIC2 primers (5′-AAGTAAGTGACTGGGGTGAGC-3′) as described by Stehling.

et al. [23]. The ERIC-PCR was carried out in a 50 µL reaction containing 25 µL of 2X Taq DNA Polymerase Master Mix RED (AMPLIQON, Co., Denmark), 1 µL of each primer, 2 µL DNA templates, and 21 µL DNase and RNase-free sterile water (Tehran Cavosh Clon, Co., Iran) in a thermal cycler (Biometra, Co., Germany). PCR amplification was done at 95 °C for 5 min for DNA denaturation, 35 cycles at 95 °C for 60 s, 56 °C for 60 s for annealing, and extension at 72 °C for 4 min, and finally 72 °C for 10 min. The PCR products were separated by electrophoresis in a 1% agarose gel in TBE 0.5x, and the gels were analyzed by Dice + UPGMA analysis with 0.2% distance using http://insilico.ehu.eus/dice_upgma/ for discrimination of the isolates, definition of clusters, and interpretation of the dendrogram.

### *Statistical analysis of* results

For statistical analysis of results, we used GraphPad Prism 8 software (GraphPad Software Inc., USA). Two-sided Student’s t-test and ANOVA tests were used to evaluate the transcript levels of *mexA*, *oprD*, and *ampC* in comparison to *P. aeruginosa* PAO1 as a calibrator. For the MIC and MBIC, all data were first assessed for normality using a Kolmogorov-Smirnov test. The results were found to be normally distributed (*p* > 0.05 in the K-S test) and were analyzed using a one-way ANOVA test and expressed as mean values ± standard (mean ± SEM). Pairwise comparisons between groups were then made using Tukey’s post hoc tests, where the main effect was seen in ANOVA tests. Data that were not normally distributed (*p* < 0.05 in the K-S test) were analyzed using a Kruskal-Wallis test. Where the main effect was seen in Kruskal-Wallis tests, pairwise comparisons between groups were made using Dunn’s multiple comparisons test. In each case, *p* ≤ 0.05 was considered statistically significant.

## Results

**Fig. 1:**
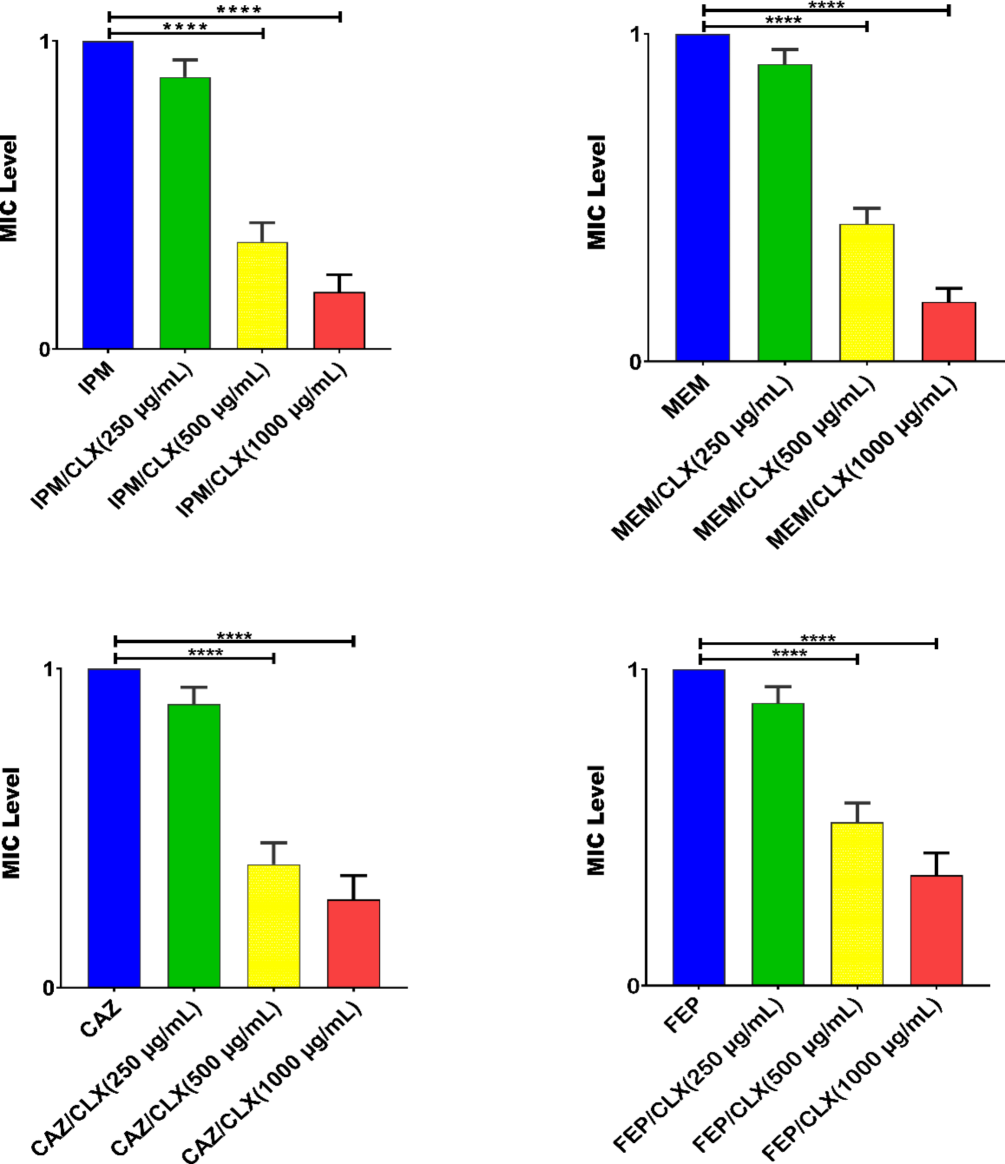
Effects of cloxacillin (CLX: 250, 500, and 1000 µg/mL) on the levels of minimum inhibitory concentration (MIC; µg/mL) of imipenem (IPM), meropenem (MEM), ceftazidime (CAZ), and cefepime (FEP) on carbapenem-resistant *P. aeruginosa* (CRPA). Graphs were drawn based on fold changes of MIC of IPM, MEM, CAZ, and FEP in combination with CLX. Data are displayed as the mean ± standard error of the mean from three replicate experiments and were analyzed using ANOVA and the nonparametric Kruskal-Wallis tests. CLX at 500 and 1000 µg/mL significantly decreased the level MIC in isolates to IPM, MEM, CAZ, and FEP. ****, statistical significance with *p* ≤ 0.0001

**Fig. 2:**
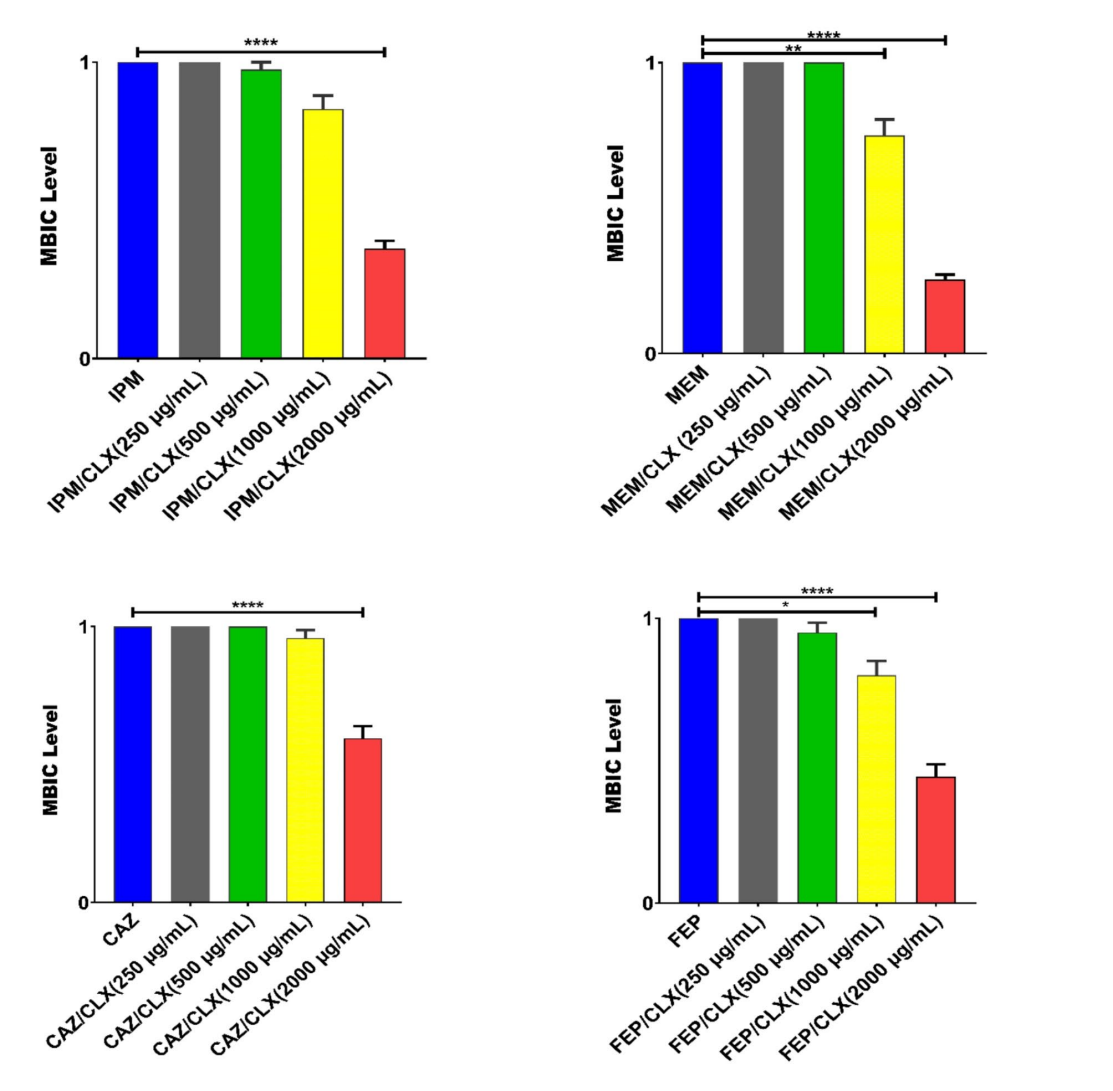
Effects of cloxacillin (CLX: 250, 500, 1000, and 2000 µg/mL) on the levels of minimal biofilm inhibitory concentration (MBIC; µg/mL) of imipenem (IPM), meropenem (MEM), ceftazidime (CAZ), and cefepime (FEP) on carbapenem-resistant *P. aeruginosa* (CRPA). Graphs were drawn based on fold changes of MBIC of IPM, MEM, CAZ, and FEP in combination with CLX. Data are displayed as the mean ± standard error of the mean from 3 replicate experiments and were analyzed using ANOVA and the nonparametric Kruskal-Wallis tests. CLX at 1000 and 2000 µg/mL significantly decreased the MBIC of isolates to MEM and FEP, and at 2000 µg/mL to IPM and CAZ. *, statistical significance with *p* ≤ 0.05, **, statistical significance with *p* ≤ 0.01, and ****, statistical significance with *p* ≤ 0.0001

**Fig. 3:**
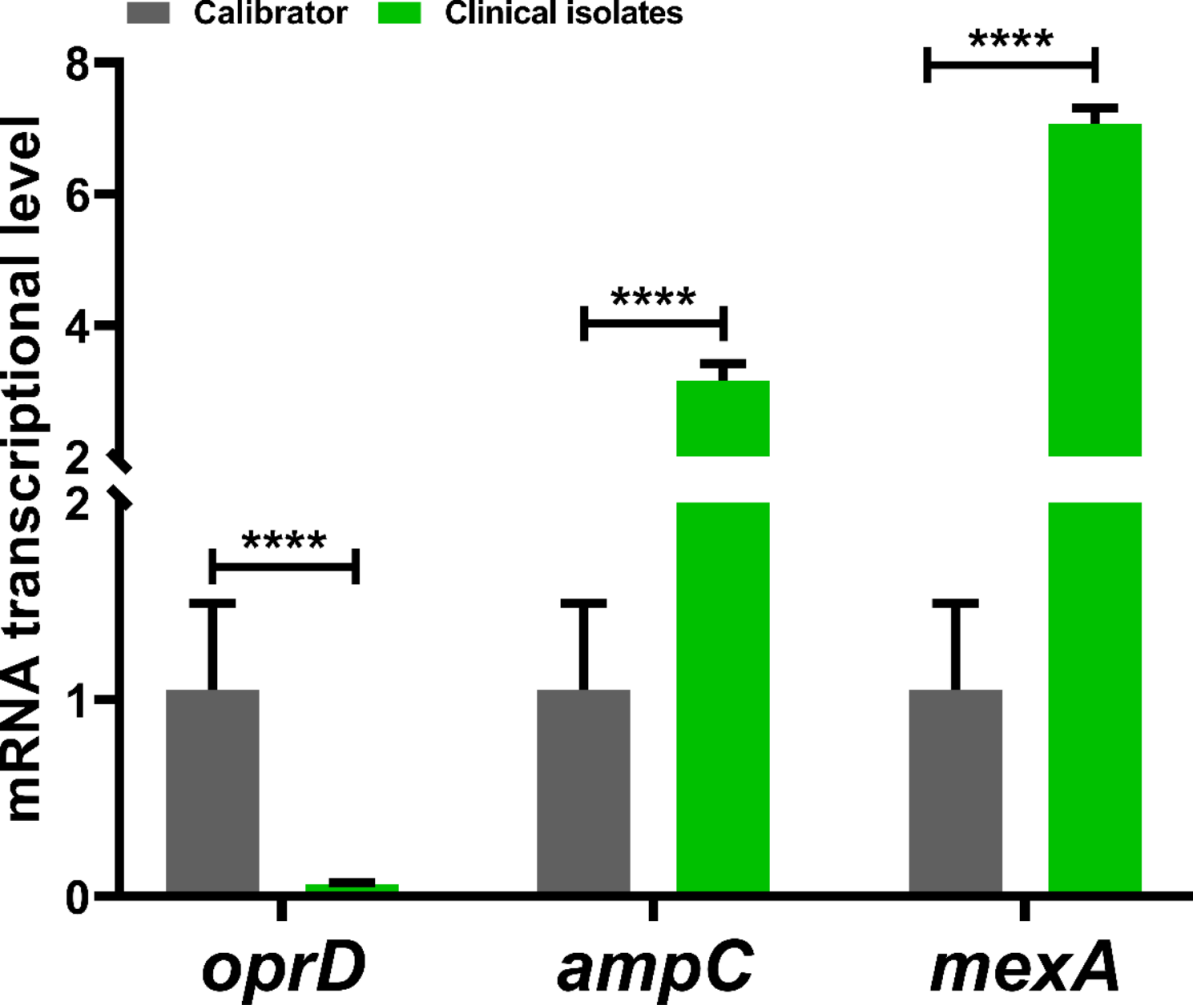
The transcriptional level of *oprD*, *ampC*, and *mexA* in carbapenem-resistant *P. aeruginosa* (CRPA) compared to PAO1. Graphs were drawn based on fold changes in transcriptional levels of *oprD*, *ampC*, and *mexA* in clinical isolates. Data are displayed as the mean ± standard error of the mean from three replicate experiments and were analyzed using the ANOVA test. ****, statistical significance with *p* ≤ 0.0001

**Fig. 4:**
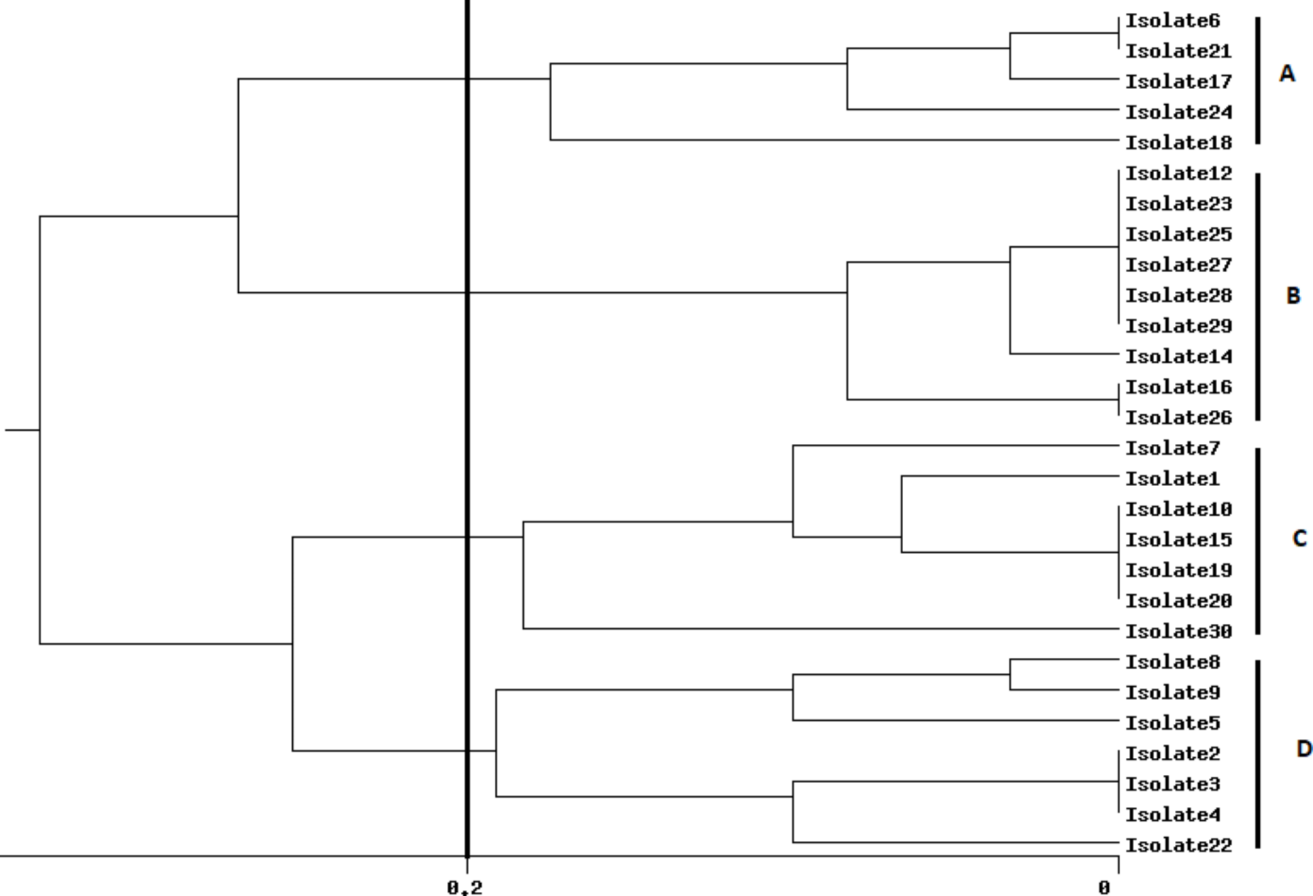
ERIC-PCR analysis of genetic variation among 30 carbapenem-resistant *P. aeruginosa* isolates. Dendrogram computed by the Dice + UPGMA analysis method with 0.2% distance

This study found that all isolates were resistant to multiple antibiotics, including IPM, MEM, FEP, CAZ, GEN, TOB, and CIP, using different mechanisms of resistance as well as exhibiting high levels of biofilm formation. The MIC levels of antibiotic agents for isolates were shown in Table 2. Twenty-seven (90%) of the isolates were considered as AmpC overproducers using antibiotic agents in combination with 250 and 500 µg/mL of CLX (Tables 2 and 3). These findings showed AmpC production is one of the main mechanisms of resistance to β-lactam antibiotics among our carbapenem-resistant isolates because CLX as an AmpC β-lactamase inhibitor MIC level to IPM, MEM, FEP, and CAZ decreased to 2-512-fold (Table 3). The level and fold change of MIC to IPM, MEM, FEP, and CAZ with/without different concentrations of CLX among the isolates are presented in Tables 3 and 4. In this study, CLX in concentrations of 500 and 1000 µg/mL significantly (*p* < 0.0001) decreased the level of MICs to IPM, MEM, CAZ, and FEP (Table 3). In the presence of 250 µg/mL of CLX only in 4 (13.3%) isolates the level of MIC to IPM, MEM, CAZ, and FEP 2-16-fold decreased but the fold change of MIC level to IPM, MEM, CAZ, and FEP in the presence of 500 and 1000 µg/mL of CLX (2-64-fold) decreased (Tables 3 and 4; Fig. 1). All of the isolates were strong biofilm producers, and the MBIC of isolates for IPM, MEM, CAZ, and FEP in combination with 2000 µg/mL of CLX significantly decreased 2-4-fold (Tables 3 and 5). The MBIC of isolates to IPM, MEM, CAZ, and FEP had not changed in the presence of 250 and 500 µg/mL of CLX (Tables 3 and 5, and Fig. 2). The carbapenemase resistance genes, including *bla*_NDM_, *bl*a_IMP_, *bla*_VIM_, *bla*_SIM_, and *bla*_GES_, were detected in 21 (70%), 6 (20%), 3 (10%), 2 (6.6%), and 1 (3.3%) of the isolates, respectively. Twenty-eight (93.3%) of the isolates were positive for one of the carbapenemase genes. Among the 16S rRNA methylase genes that were evaluated in this study, only *armA* was observed in 17 (56.6%) of isolates, and the MIC level to GEN was higher in isolates with *armA* compared to isolates that were negative for *armA*. The most prevalent co-presence of resistance genes was *bla*_NDM_+*armA* in 9 (30%), followed by *bla*_IMP_+*armA* in 3 (10%), *bla*_NDM_+*bla*_IMP_ in 2 (6.6%), *bla*_NDM_+*bla*_SIM_+*armA* in 2 (6.6%), *bla*_NDM_+*bla*_VIM_+*armA* in 1 (3.3%), and *bla*_NDM_+*bla*_GES_+*armA in* 1 (3.3%) of the isolates (Table 2). The qPCR experiment revealed the transcriptional level of *oprD* was significantly 10-fold downregulated in clinical isolates compared with the PAO1 strain as a calibrator. Also, the expression levels of *ampC* and *mexA* were increased 2-4-fold and 2-8-fold in compared with PAO1, respectively (Fig. 3). According to ERIC-PCR typing results among 30 clinical isolates of *P. aeruginosa*, 30 isolates were divided into 4 clusters (A to D), while two isolates were non-typeable by ERIC-PCR (Fig. 4). Five (16.6%) isolates were in cluster A, 9 (30%) isolates in cluster B, 7 (23.3%) isolates in cluster C, and 7 (23.3%) isolates in cluster D (Fig. 4). No significant relationship was observed between clusters (A to D) with the profile of resistance genes and MIC levels of isolates to antibiotic agents, which has been probably due to the heterogeneity of the isolates and genetic exchange between isolates in hospital settings. The distributions of ERIC types with profiles of resistance genes and MIC levels were shown in Table 2.

**Table 2:** The characterization of ERIC types, MIC levels (µg/mL) to antibiotic agents, AmpC phenotype, and profile of resistance genes among CRPA isolates

Isolates	ERIC-PCR type	MIC						ApmC	Resistance genes profile
		IPM	MEM	CAZ	FEP	GEN	CIP		
1	C	16	16	64	32	8	4	+	-
2	D	16	32	64	64	8	8	+	*bla*_VIM_
3	D	32	64	256	64	1024	4	+	*bla*_IMP_, and *armA*
4	D	32	64	256	64	128	8	+	-
5	D	32	64	64	64	16	8	+	*bla*_NDM_
6	A	256	≥ 2048	≥ 4096	512	16	32	+	*bla*_NDM_, and *armA*
7	C	64	64	128	64	64	16	-	*bla*_NDM_
8	D	32	64	≥ 4096	512	512	8	+	*bla*_NDM,_ and *bla*_IMP_
9	D	64	64	128	64	128	8	+	*bla*_NDM_
10	C	256	≥ 2048	≥ 4096	512	64	16	-	*bla*_NDM,_ and *bla*_IMP_
11	NT	128	64	256	128	64	16	+	*bla*_NDM_, *bla*_SIM_, and *armA*
12	B	64	128	128	128	1024	32	+	*bla*_IMP_, and *armA*
13	NT	64	64	128	64	64	16	+	*bla*_NDM_, and *armA*
14	B	64	64	128	64	64	16	+	*bla*_NDM_, and *armA*
15	C	128	512	128	256	64	8	+	*bla*_NDM_, and *armA*
16	B	32	128	64	32	64	32	+	*bla*_NDM_, and *armA*
17	A	64	64	128	64	64	16	+	*bla*_NDM_, and *armA*
18	A	64	128	512	64	128	4	+	*bla*_NDM_, and *armA*
19	C	64	64	128	64	64	16	+	*bla*_IMP_, and *armA*
20	C	32	64	64	64	16	8	+	*bla*_NDM_
21	A	32	64	64	64	16	8	+	*bla*_NDM_
22	D	64	64	128	64	16	16	+	*bla*_IMP_
23	B	128	64	256	128	16	16	+	*bla*_NDM_, *bla*_GES_, and *armA*
24	A	32	64	64	64	16	8	+	*bla*_NDM_
25	B	64	128	64	64	32	64	+	*bla*_NDM_, and *armA*
26	B	128	128	512	256	64	16	+	*bla*_NDM_, *bla*_VIM_, and *armA*
27	B	16	16	64	64	64	8	+	*armA*
28	B	64	128	64	64	32	64	+	*bla*_NDM_, and *armA*
29	B	128	64	256	128	64	16	-	*bla*_NDM_, *bla*_SIM_, and *armA*
30	C	32	16	128	64	16	8	+	*bla*_VIM_

**IPM**: Imipenem, **MEM**: Meropenem, **CAZ**: Ceftazidime, **FEP**: Cefepime, **GEN**: Gentamicin, **CIP**: Ciprofloxacin, **CRPA**: Carbapenem-resistant *P. aeruginosa*, **NT**: Non-typeable

**Table 3:** The ranges and fold changes of the MIC and MBIC of IPM, MEM, CAZ, and FEP with/without CLX among CRPA isolates

MIC (µg/mL)						MBIC (µg/mL)					
Antibiotic agents	MIC_50/90_	MIC Range	MIC Fold Changes	Isolates *n* (%)	*p*-Value	Antibiotic agents	MBIC_50/90_	MBIC Range	MBIC Fold Changes	Isolates *n* (%)	*p*-Value
**IPM**	**64/128**	**16–256**	**-**		**-**	**IPM**	**128/512**	**64–512**	**-**	-	**-**
IPM + CLX (250 µg/mL)	64/128	2-256	8↓	4(12)	> 0.9999	IPM + CLX (250 and 500 µg/mL)	128/512	64–512	0	1(3)	> 0.9999
IPM + CLX (500 µg/mL)	16/64	≤ 0.5–256	2–32↓	25(75)	< 0.0001	IPM + CLX (1000 µg/mL)	128/512	32–512	2↓	8(24)	> 0.0688
IPM + CLX (1000 µg/mL)	2/32	≤ 0.5–256	2-256↓	27(81)	< 0.0001	IPM + CLX (2000 µg/mL)	32/256	16–256	2–4↓	30(100)	< 0.0001
**MEM**	**64/512**	**16-2048**	**-**		**-**	**MEM**	**256/1024**	**128–4096**	**-**	-	**-**
MEM + CLX (250 µg/mL)	64/128	8-2048	2–4↓	4(12)	> 0.9999	MEM + CLX (250 and 500 µg/mL)	256/1024	128–4096	0	0(0)	> 0.9999
MEM + CLX (500 µg/mL)	32/64	2-1024	2–16↓	27(81)	< 0.0001	MEM + CLX (1000 µg/mL)	256/1024	64-2048	2–4↓	13(39)	0.0060
MEM + CLX (1000 µg/mL)	8/64	0.5–1024	2-256↓	29(87)	< 0.0001	MEM + CLX (2000 µg/mL)	128/512	32-1024	2–4↓	30(100)	< 0.0001
**CAZ**	**128/512**	**64-4096**			**-**	**CAZ**	**512/1024**	**256–8192**	**-**	**30(100)**	**-**
CAZ + CLX (250 µg/mL)	128/512	8-4096	2–16↓	4(12)	> 0.9999	CAZ + CLX (250 and 500 µg/mL)	512/1024	128–4096	0	0(0)	> 0.9999
CAZ + CLX (500 µg/mL)	32/256	2-4096	2–64↓	23(69)	< 0.0001	CAZ + CLX (1000 µg/mL)	512/1024	64-4096	2↓	7(21)	> 0.9999
CAZ + CLX (1000 µg/mL)	4/256	0.5–4096	64-1024↓	23(69)	< 0.0001	CAZ + CLX (2000 µg/mL)	256/1024	64-4096	2–4↓	21(63)	< 0.0001
**FEP**	**64/512**	**32–512**			**-**	**FEP**	**256/1024**	**128–1024**	**-**	30(100)	**-**
FEP + CLX (250 µg/mL)	64/256	8-512	4–16↓	4(12)	> 0.9267	FEP + CLX (250 and 500 µg/mL)	256/1024	128–1024	4↓	2(6)	> 0.9999
FEP + CLX (500 µg/mL)	32/256	2-512	2–64↓	22(66)	< 0.0001	FEP + CLX (1000 µg/mL)	256/512	64-1024	2–4↓	9(27)	> 0.0112
FEP + CLX (1000 µg/mL)	16/128	0.5–512	4-512↓	23(69)	< 0.0001	FEP + CLX (2000 µg/mL)	128/256	32-1024	2–4↓	27(81)	0.0001

**MIC**: Minimum Inhibitory Concentration, **MBIC**: Minimal Biofilm Inhibitory Concentration, **CLX**: Cloxacillin, **IPM**: Imipenem, **MEM**: Meropenem, **CAZ**: Ceftazidime, and **FEP**: Cefepime, and **CRPA**: Carbapenem-resistant *P. aeruginosa*

**Table 4:** The MIC level (µg/mL) of IPM, MEM, CAZ, and FEP with/without 250, 500, and 1000 µg/mL of CLX among CRPA isolates

Isolates	IPM	IPM/ 250 CLX	IPM/ 500 CLX	IPM/ 1000 CLX	MEM	MEM/ 250 CLX	MEM/ 500 CLX	MEM/ 1000 CLX	CAZ	CAZ/ 250 CLX	CAZ/ 500 CLX	CAZ/ 1000 CLX	FEP	FEP/ 250 CLX	FEP/ 500 CLX	FEP/ 1000 CLX
1	16	2	≤ 0.5	≤ 0.5	16	8	2	≤ 0.5	64	16	8	≤ 0.5	32	8	2	≤ 0.5
2	16	2	≤ 0.5	≤ 0.5	32	8	2	≤ 0.5	64	16	8	≤ 0.5	64	16	8	≤ 0.5
3	32	32	≤ 0.5	≤ 0.5	64	64	16	≤ 0.5	256	256	128	32	64	64	16	8
4	32	32	4	≤ 0.5	64	64	64	32	256	256	256	256	64	64	64	64
5	32	32	16	4	64	64	32	4	64	64	16	2	64	64	32	4
6	256	256	256	256	≥ 2048	≥ 2048	1024	512	≥ 4096	≥ 4096	≥ 4096	≥ 4096	512	512	512	512
7	64	64	16	2	64	64	16	4	128	128	32	16	64	64	32	16
8	32	32	16	8	64	64	32	16	≥ 4096	≥ 4096	2048	1024	512	512	256	32
9	64	64	64	32	64	64	64	64	128	128	128	128	64	64	64	64
10	256	256	256	256	≥ 2048	≥ 2048	1024	1024	≥ 4096	≥ 4096	≥ 4096	≥ 4096	512	512	512	512
11	128	128	16	2	64	64	32	4	256	256	64	32	128	128	128	64
12	64	64	64	32	128	128	64	32	128	128	32	16	128	128	128	128
13	64	8	2	≤ 0.5	64	16	8	≤ 0.5	128	8	2	≤ 0.5	64	16	2	≤ 0.5
14	64	64	16	2	64	64	32	4	128	128	64	16	64	64	32	16
15	128	128	64	32	512	512	256	128	128	128	128	128	256	256	256	256
16	32	32	32	32	128	128	64	64	64	64	64	64	32	32	16	8
17	64	64	32	8	64	64	16	4	128	128	32	4	64	64	32	4
18	64	64	32	8	128	128	64	32	512	512	64	32	64	64	32	32
19	64	64	16	4	64	64	32	8	128	128	8	2	64	64	32	16
20	32	32	4	≤ 0.5	64	64	32	8	64	64	16	2	64	64	32	8
21	32	32	4	≤ 0.5	64	64	32	8	64	64	16	4	64	64	32	16
22	64	64	4	≤ 0.5	64	64	32	8	128	128	16	4	64	64	32	16
23	128	128	16	2	64	64	32	8	256	256	32	4	128	128	32	16
24	32	32	4	≤ 0.5	64	64	16	2	64	64	8	1	64	64	16	8
25	64	64	32	16	128	128	128	64	64	64	64	64	64	64	64	64
26	128	16	4	≤ 0.5	128	32	8	≤ 0.5	512	64	16	≤ 0.5	256	16	4	≤ 0.5
27	16	16	2	≤ 0.5	16	16	4	1	64	64	8	2	64	64	16	4
28	64	64	16	2	128	128	32	8	64	64	8	2	64	64	16	4
29	128	128	16	2	64	64	8	2	256	256	32	8	128	128	32	8
30	32	32	8	1	16	16	2	≤ 0.5	128	128	16	2	64	64	16	4

**MIC**: Minimum Inhibitory Concentration, **CLX**: Cloxacillin, **IPM**: Imipenem, **MEM**: Meropenem, **CAZ**: Ceftazidime, and **FEP**: Cefepime, and **CRPA**: Carbapenem-resistant *P. aeruginosa*

**Table 5:** The MBIC level (µg/mL) of IPM, MEM, CAZ, and FEP with/without 250, 500, 1000, and 2000 µg/mL of CLX among CRPA isolates

Isolates	IPM	IPM/ 250 CLX	IPM/ 500 CLX	IPM/ 1000 CLX	IPM/ 2000 CLX	MEM	MEM/ 250 CLX	MEM/ 500 CLX	MEM/ 1000 CLX	MEM/ 2000 CLX	CAZ	CAZ/ 250 CLX	CAZ/ 500 CLX	CAZ/ 1000 CLX	CAZ/ 2000 CLX	FEP	FEP/ 250 CLX	FEP/ 500 CLX	FEP/ 1000 CLX	FEP/ 2000 CLX
1	64	64	64	64	32	256	256	256	256	64	512	512	512	512	256	256	256	256	256	128
2	64	64	64	64	32	256	256	256	256	64	512	512	512	512	256	256	256	256	256	128
3	128	128	128	128	64	128	128	128	64	32	1024	1024	1024	1024	512	128	128	128	128	64
4	64	64	64	64	32	256	256	256	256	64	512	512	512	512	256	256	256	256	256	128
5	64	64	64	64	32	256	256	256	256	64	512	512	512	512	256	256	256	256	256	128
6	512	512	512	512	256	≥ 4096	≥ 4096	≥ 4096	2048	1024	≥ 4096	≥ 4096	≥ 4096	≥ 4096	≥ 4096	1024	1024	1024	1024	1024
7	128	128	128	64	32	1024	1024	1024	1024	512	128	128	128	128	64	128	128	128	64	32
8	64	64	64	64	32	128	128	128	64	32	≥ 4096	≥ 4096	≥ 4096	≥ 4096	2048	1024	1024	1024	512	64
9	512	512	512	512	256	512	512	512	512	256	1024	1024	1024	1024	1024	1024	1024	1024	1024	1024
10	512	512	512	512	256	≥ 4096	≥ 4096	≥ 4096	2048	1024	≥ 4096	≥ 4096	≥ 4096	≥ 4096	≥ 4096	1024	1024	1024	1024	1024
11	512	512	512	512	128	1024	1024	1024	1024	256	1024	1024	1024	1024	512	512	512	512	512	256
12	128	128	128	64	32	512	512	512	256	128	1024	1024	1024	1024	256	512	512	512	256	128
13	64	64	64	64	32	256	256	256	256	64	512	512	512	512	256	256	256	256	256	128
14	128	128	128	128	64	128	128	128	64	32	1024	1024	1024	1024	512	128	128	128	128	64
15	512	512	512	512	128	1024	1024	1024	1024	256	1024	1024	1024	1024	512	512	512	512	512	256
16	128	128	128	128	32	256	256	256	256	64	256	256	256	256	256	128	128	128	128	64
17	128	128	128	128	64	256	256	256	256	64	512	512	512	128	64	512	512	128	128	64
18	128	128	128	64	16	256	256	256	128	32	1024	1024	1024	1024	512	128	128	128	64	32
19	128	128	128	128	64	128	128	128	64	32	1024	1024	1024	1024	512	128	128	128	128	64
20	64	64	64	32	16	256	256	256	64	32	512	512	512	256	256	256	256	256	128	64
21	64	64	64	32	16	256	256	256	64	32	512	512	512	256	256	256	256	256	128	64
22	64	64	64	32	16	256	256	256	64	32	512	512	512	256	256	256	256	256	128	64
23	512	512	512	512	128	1024	1024	1024	1024	256	1024	1024	1024	1024	512	512	512	512	512	256
24	64	64	64	32	16	256	256	256	64	32	512	512	512	256	256	256	256	256	128	64
25	256	256	256	128	32	512	512	512	512	128	256	256	256	256	128	256	256	256	256	128
26	256	256	64	64	32	256	256	256	256	128	1024	1024	1024	512	256	512	512	512	256	128
27	64	64	64	64	32	256	256	256	256	64	512	512	512	512	256	256	256	256	256	128
28	128	128	128	128	64	512	512	512	256	128	256	256	256	64	64	512	512	128	128	64
29	512	512	512	512	128	1024	1024	1024	1024	256	1024	1024	1024	1024	512	512	512	512	512	256
30	64	64	64	64	32	256	256	256	256	64	512	512	512	512	256	256	256	256	256	128

**MBIC**: Minimal biofilm inhibitory concentration, **CLX**: Cloxacillin, **IPM**: Imipenem, **MEM**: Meropenem, **CAZ**: Ceftazidime, **FEP**: Cefepime, and **CRPA**: Carbapenem-resistant *P. aeruginosa*

## Discussion

*P. aeruginosa* is an important opportunistic bacterium that is implicated in serious nosocomial infections such as pneumonia and sepsis [22]. Carbapenems are the most important options in the treatment of hospital infections caused by MDR *P. aeruginosa* [24]. Regrettably, there is a growing prevalence of infections caused by this bacterium that their treatment with carbapenems has failed [16]. Several mechanisms, including AmpC overproduction, the production of carbapenemases, the overactivity of efflux pumps, and the loss or downregulation of outer membrane porins such as OprD, are involved in carbapenem resistance in *P. aeruginosa*. On the other hand, according to some reports, carbapenem-resistant bacteria are usually resistant to other classes of antibiotic agents, such as fluoroquinolones and aminoglycosides [25]. During the past decade, one of the main mechanisms of resistance in gram-negative bacilli that could be coupled with carbapenemase genes is resistance to aminoglycosides by 16S rRNA methylase genes such as *armA*, *rmtA* to *rmtH*, and *npmA* [26]. Risk factors, including long hospitalizations, invasive medical interventions, and prolonged antibiotic therapy, are the most important factors for developing antimicrobial resistance mechanisms in hospital settings [27].

This study aimed to evaluate the mechanism of resistance to carbapenems and aminoglycosides as well as the effect of CLX on the antibacterial and antibiofilm activity of CAZ, FEP, IMP, and MEM against carbapenem-resistant *P. aeruginosa* (CRPA) clinical isolates.

Global reports indicated that the prevalence of CRPA depends on multiple factors such as geographic region, infection type and microorganism, specimen source, antibiotic selective pressure, and quality of hygiene in the environment in hospital settings [28]. However, the rate of carbapenem resistance was reported between 10 and 100% in different countries [29–32]. In Iran, rates of resistance to carbapenems in clinical isolates of *P. aeruginosa* are different, which can be due to the vast geography of our country or different treatment policies. For example, Mirsalehian et al.‘s studies in Tehran, Iran, showed that 100% of the *P. aeruginosa* from wounds of burn patients were carbapenem-resistant, and Farajzadeh Sheikh et al. showed that 90.7% of *P. aeruginosa* isolates from Ahvaz in southwest Iran were resistant to carbapenems [7, 33]. Also, resistance to carbapenems in Kerman, Iran, was %35.8 in 2020; the results of the present study show an increase in resistance to carbapenems compared to the past in our region [15].

In our study, the prevalence of resistance genes among the isolates was 63% for *bla*_NDM_, 56.6% for *armA*, 18% for *bla*_IMP_, 9% for *bla*_VIM_, 3% for *bla*_GES_, and 6% for *bla*_SIM_, and unfortunately, in most cases, these carbapenemase genes were observed stimulatingly with *armA* as a 16S rRNA methylase that caused high-level resistance to aminoglycosides. So, the spread of the clinical isolates that co-harbor carbapenemase and 16S rRNA methylase genes are a potential risk in reducing treatment options and failure to treat infections. In addition, these isolates can play an important role as a reservoir of resistance genes and the transfer of them to other bacteria [34].

Co-existence of *bla*_NDM_/16S rRNA methylase genes with a prevalence of 21.14% was reported for the first time in 2018 among clinical isolates of *Klebsiella pneumoniae* in our region, and then in 2020, a case of *P. aeruginosa* isolate with *bl*a_NDM_ was reported [15, 35]. So, our finding in the present study shows an increase in the *bl*a_NDM_ among *P. aeruginosa* isolates compared to 2020, which indicates the lack of appropriate strategies to control infection and the possible horizontal transfer of this gene among different bacteria in hospital settings. Since probably the genetic transfer of carbapenemase and 16S rRNA methylase genes among bacteria in our hospital is related to each other, they may develop into endemic resistance genes in this area, although further research is needed in this regard. In any case, the spread of such isolates is a serious concern in public health, and we need an appropriate strategy for epidemiological surveillance, comprehensive infection control, and antibiotic therapy. Also, in recent years, isolates that have both carbapenemase and 16S rRNA methylase, such as *bla*_NDM−1_+*rmtC*, *bla*_NDM−1_+*rmtC* + *rmtF*, *bla*_NDM−1_+*rmtF* + *armA*, and *bla*_NDM−1_+*bla*_VIM_+*rmtB*, have been reported in many countries, especially Iraq and Pakistan as our neighboring countries [33, 35–37].

Other mechanisms that are associated with resistance to carbapenems in *P. aeruginosa* are downregulation of *oprD*, overproduction of AmpC β-lactamase, and overexpression of efflux pumps such as *mexAB*-*oprM*, all of which were observed among carbapenem resistance isolates in this study.

In this study, *oprD* transcription levels in clinical isolates were significantly reduced 10-fold compared to the PAO1 strain, and the expression levels of *ampC* and *mexA* were increased by 2-4-fold and 2-8-fold, respectively, compared to PAO1.

In a study by Rostami et al. (2018) in Iran, the most common mechanisms of resistance to carbapenem among *P. aeruginosa* isolates were overexpression of the *mexB*, *mexY*, *ampC*, and downregulation of *oprD* with a range of MIC 16-≥256 µg/mL to IPM or MEM among them, and only 18% of isolates were positive for *bla*_VIM_ and *bla*_IMP_ [38]. In our study, in contrast to Rostami et al., 90% of the isolates were positive for one of the carbapenemase genes, including *bla*_NDM_, *bla*_IMP_, *bla*_SIM_, *bl*a_VIM_, and *bla*_GES_ that were coupled with overexpression of *mexA* and *ampC*, so the MIC range in the present study (16–2048 µg/mL to IPM or MEM) was higher.

Cabot et al. have demonstrated that *ampC* overexpression was the major resistance mechanism in 190 *P. aeruginosa* isolates obtained from bloodstream infections, and other studies show that the main resistance mechanisms to imipenem among *P. aeruginosa* in China are loss of *oprD* or production of MBLs [24, 39]. Therefore, similar to the current study, usually different mechanisms are involved in carbapenem resistance in *P. aeruginosa*. This simultaneous existence of carbapenem resistance mechanisms, as well as *armA*, have played a significant role in reducing susceptibility to carbapenems and treatment failure among *P. aeruginosa*.

In the current study, we evaluated the effects of cloxacillin (CLX) on the MIC and MBIC of isolates to MEM, CAZ, IMP, and FEP. The MIC level to MEM, IPM, CAZ, and FEP significantly decreased at 500 and 1000 µg/mL of CLX, and the reduction of MBIC to MEM, IPM, CAZ, and FEP was significantly observed at 1000 and 2000 µg/mL of CLX among the isolates. Pehlvanzadeh et al. have shown that the combination of IPM, MEM, CAZ, and FEP with 250 µg/mL CLX leads to a significant reduction in MIC level in 56% of carbapenem-resistant *P. aeruginosa* isolates [16]. Also, Rodríguez et al. (2010) observed that among *P. aeruginosa*, the MIC level reduced 2-32-fold to IPM in 84% of isolates and 2-8-fold to CAZ in 78% of isolates in the presence of 250 µg/mL of CLX [18]. Mirsalehian et al. reported 2-8-fold decreasing of MIC to IPM and 1-2-fold to CAZ in 86% and 9% of carbapenem-resistant *P. aeruginosa*, respectively, at 250 µg/mL of CLX [40]. Several reasons can cause a difference in decreasing susceptibility of isolates to IPM, MEM, CAZ, and FEP in the presence of 500 and 1000 µg/mL of CLX in our study compared to previous studies that showed significant reducing of MIC to IPM, MEM, CAZ, and FEP in the presence of 250 µg/mL CLX. In our study, more than 90% of isolates had at least one carbapenemase gene, but in Pahlavanzadeh and Mirsalehian’s studies, this prevalence of carbapenemase genes was not reported [7, 16]. Also, some point mutations in the *ampC* gene, especially substitution in the alanine residue at position 105, upregulation of it, and intrinsic resistance to CLX in *P. aeruginosa*, such as the low membrane permeability, efflux pump activity, and hydrolysis of CLX by *bla*_OXA_, can reduce the effect or affinity of CLX on the AmpC β-lactamase or *P. aeruginosa* [18]. On the other hand, according to our finding, the effective concentration of cloxacillin to reduce the MIC of isolates to CAZ, FEP, IPM, and MEM is ≥ 500 µg/mL, which is much higher than the serum level of cloxacillin in humans. Therefore, the presence of different mechanisms of resistance to β-lactams in *P. aeruginosa* can have an effect on the ability of CLX to inhibit AmpC β-lactamase, and a combination of antipseudomonal β-lactams with CLX may not be appropriate or useful to the treatment of infections by *P. aeruginosa*.

In addition to antibiotic resistance, biofilm formation in bacteria causes recurrence of infection and increases pathogenicity [13]. All *P. aeruginosa* isolates in this study, similar to other reports, have been identified as strong biofilm producers [41]. The MIC level was significantly reduced to IMP, MEM, CAZ, and FEP in the presence of 250–500 µg/mL of CLX in our study and other reports, but the MBIC level to IMP, MEM, CAZ, and FEP decreased in the presence of 2000 µg/mL of CLX in this study. Therefore, it seems that overexpression or inhibition of AmpC β-lactamase does not play an important role in biofilm formation, although some studies have shown upregulation of *ampC* genes in the biofilm form of *P. aeruginosa* [42]. Also, among the β-lactam antibiotics in this study, IPM was the most effective on the MBIC level with/without CLX, which represents more inhibitory effects of IPM on biofilm formation than other β-lactam antibiotics.

Biofilm formation depends on various environmental and genetic factors [43]. One of the effective factors in the formation of biofilm is the increase in the activity or upregulation of efflux pumps, which is probably due to the secretion of quorum sensing mediators by them; in the present study, all of the isolates were *mexA* overexpression [43, 44]. Also, according to some studies, upregulation of efflux pumps and *ampC* is usually coupled with downregulation of outer membrane proteins such as OprD, which has an important role for resistance to carbapenem antibiotics. In this study, we observed upregulation of *mexA* and *ampC* and downregulation of the *oprD* [45]. However, the simultaneous presence of several antibiotic resistance mechanisms, as well as biofilm formation, plays a main role in increasing carbapenem resistance.

In ERIC-PCR typing, the isolates were divided into 4 clusters from A to D, while two isolates were non-typeable, which revealed that isolates with the *bla*_NDM_ may belong to different ERIC-types and vice versa. These findings showed genomic heterogeneity among the isolates that harbored different resistance genes and MIC levels to antibiotic agents (see Table 2; Fig. 4). The genetic diversity indicated that our isolates were not from an outbreak of a single clone, so we suggest that *bla*_NDM_-positive isolates with different ERIC-types may have increased their epidemic potential that may be associated with horizontal gene transfer (HGT) or chromosomally mutations and can potentially play a significant role in the dissemination of resistance genes in our hospital settings. Also, we did not find any significant relationship between antibiotic resistance pattern, MIC level, AmpC overproduction, and presence of resistance genes with clusters A to D, which could be due to the genetic heterogeneity, low number of isolates, and horizontal gene transfer among our isolates. However, this finding shows the spread of high-level resistant *P. aeruginosa* isolates to different antibiotics in our hospital settings, and we need to use more appropriate policies in the infection control program.

## Conclusion

Different intrinsic and acquired resistance mechanisms, as well as biofilm formation, are responsible for resistance to carbapenems and aminoglycosides in *P. aeruginosa* isolates in Kerman, Iran. Our study revealed that, in addition to carbapenemase genes, particularly *bla*_NDM_, other mechanisms such as *oprD* downregulation, AmpC overproduction, and increased expression of the *mexAB-oprM* efflux system have contributed to the emergence of high-level resistance isolates against carbapenems. Also, for the first time, we reported carbapenem resistance in *P. aeruginosa* isolates harboring *bla*_NDM_, *bla*_SIM_, *bla*_GES_, *bla*_VIM_, and *bla*_IMP_ with *armA* 16S rRNA methylase genes, demonstrating the endemic dissemination of these genes in our hospital. These findings highlight limited options for antimicrobial therapy of patients who are infected with carbapenem-/multidrug-resistant *P. aeruginosa* isolates. Therefore, it is important to implement genomic surveillance in low-resource settings to promptly identify and control the spread of carbapenem resistance and monitor evolving resistant bacteria in our healthcare settings.

## Data Availability

The datasets used or analyzed during the study are available on reasonable requests from the corresponding author (E. mail: d.kalantar@kmu.ac.ir).

## Abbreviations

CRPA: Carbapenem-resistant *Pseudomonas aeruginosa*
ERIC-PCR: Enterobacterial Repetitive Intergenic Consensus Polymerase Chain Reaction
qPCR: quantitative real-time PCR
MDR: Multidrug resistant
MIC: Minimum Inhibitory Concentration
MBIC: Minimal Biofilm Inhibitory Concentration
CLX: Cloxacillin
IPM: Imipenem
MEM: Meropenem
CAZ: Ceftazidime
FEP: Cefepime
GEN: Gentamicin
TOB: Tobramycin
CIP: Ciprofloxacin
NT: Non-typeable
